# Digital health records: an essential yet neglected step in Iraq

**DOI:** 10.1093/oodh/oqaf023

**Published:** 2025-10-01

**Authors:** Ahmed A Mosa, Abdullah S Muhi, Halder J Abozait, Nawfal R Hussein

**Affiliations:** Department of Biomedical Sciences, College of Medicine, University of Zakho, Zakho Independent Administration, 42002, Kurdistan Region, Iraq; Department of Biomedical Sciences, College of Medicine, University of Zakho, Zakho Independent Administration, 42002, Kurdistan Region, Iraq; Department of Medicine, College of Medicine, University of Duhok, Duhok, Kurdistan Region, Iraq; Department of Biomedical Sciences, College of Medicine, University of Zakho, Zakho Independent Administration, 42002, Kurdistan Region, Iraq

**Keywords:** electronic health records, health information systems, health policy, patient safety, Iraq

## Abstract

It is within medicine’s nature to continuously evolve and make use of all the new technologies and advancements to enhance patient care. Digital health records have emerged as a vital tool for adhering to these recent developments. These medical databases have substantially enhanced medical practice and patient satisfaction. Yet, many low- and middle-income countries lack a foundational structure for this vital initiative. This study aims to highlight the potential advantages of implementing digital database infrastructure in Iraq, alongside exploring the possible obstacles and providing practical solutions to address those barriers. The major challenges that hinder our ability to implement a digital health system can be classified into: patient-sided barriers, provider-sided barriers and resource limitations. To overcome these barriers, our governmental efforts must encourage change through providing awareness, educational programs and resources as needed.

## BACKGROUND

Throughout its existence, medicine had always recorded patient-centered information as part of its practice, and over the years, medical information records have since evolved to incorporate recent technological advancements due to their convenience and quick access. A major transition is occurring worldwide in regards to the documentation of patient-related data within the clinical settings, as the creation of information technology and the internet has quickly accelerated the rate at which information can be stored and accessed. Digital health records provide several advantages to health care providers, patients, researchers and tailored efforts customized by local data and requirements. The system facilitated healthcare providers' access to essential patient medical information, while also empowering patients to engage actively with their treatment plans, enhancing their understanding about their illnesses, and overall, more cooperative with healthcare services, as it involved their direct participation. Thus, the transition from paper-based records to electronic records was a natural step to take [1–6].

Several governments in the low- and middle-income countries (LMICs) have not yet undertaken this vital measure due to constrained resources. Iraq can be considered one of said countries as the information management system is predominantly paper-based due to its past conflicts which heavily affected resources and management of every governmental sector, healthcare included. This led to the delay in the progression toward electronic records. It is imperative to denote that the paper-based system is inefficient and very limited in terms of accessibility to both patients and healthcare providers, it is also a rate limiter in terms of data collection and analysis, which restrict our ability to make timely use of the necessary health information [7–10].

The healthcare system within Iraq functions as a combination of public and private providers, wherein the public sector is governmentally managed and supervised through the Ministry of Health where every service is provided free of charge. While private facilities are accessible to those who can afford out-of-pocket pay [11, 12]. In Iraq, some initiatives have been established, yet there are many barriers to implement a nationwide health network. Thus, this study aims to explore the potential benefits of establishing a digital-based medical recording infrastructure in Iraq, alongside exploring the challenges and possible barriers to adopting new technologies within our health services, while providing practical solutions to address said barriers.

## THE EFFECT OF CHANGE

Many countries have reported in favor of the use of electronic medical records as they have shown a growth in patient satisfaction and communication in delivering the necessary healthcare services, maintaining a good grasp on their current and prior conditions, and overall better autonomy over their medical information and treatment regimens [5, 9, 13, 14]. A recent study shows that Australia’s implementation of electronic health records has far exceeded that of the UK and US, which allowed them to record stunning success in operative units that provided the easy access to them which encouraged a critical change in their recording system. It must also be noted that they had provided education intervals and a good infrastructure to support the learning process of their population [8, 14]. It is important to highlight that Iran, our neighboring country, had implemented a nationwide Electronic Health Record (EHR) system [15, 16]. Within the establishment of the Iranian EHR, the intended outcomes were to provide benefit to both the patients and the healthcare workers through providing an accessible, safe and easy to use system to improve quality of healthcare [16]. However, studies evaluating the success, benefits and challenges of their EHR showed that despite the intended benefits, they have experienced unsatisfying results due to the many challenges that come with Iranian EHR, most of which are related to human resources, rejection, lack of incentive to employ the EHR, dissatisfaction of the users due to the unstable nature of internet connection, and software crashing [15, 16].

Despite the continuous beneficial role that electronic records have played globally in terms of allowing faster records to be made, preventing loss or deformation of patient information, more reliable store of health data and use of said data by policymakers for effective solutions to demographic and epidemiological questions [10, 17], many countries are still hesitant toward system implementation for a variety of causes, Iraq being one of them [8, 9, 18, 19]. In Iraq, many remain unaware of such crucial systems, let alone how to implement them and those few who know were hesitant to begin familiarizing themselves with such systems due to the low potential of the implementation of digital health records [9, 10, 20].

## THE LIMITS OF OUR POTENTIAL

Several studies have investigated Iraq’s situation in terms of implementation of EHR, and multiple obstacles have been identified. The major barriers that can hinder the implementation of a digital health system can be classified into groups for the sake of simplicity: patient-sided barriers, provider-sided barriers and resource limitations [8, 9, 18, 19, 21]. The most prominent aspect of the Iraqi/Middle-Eastern culture is their reluctance in sharing personal identifiers due to their uncertainty of the information being made public and conservative nature, a potentially high presence of digital illiteracy and the lack of awareness in regards to the uses and role of digital records in older aged individuals [4, 5, 8, 9, 18, 21].

This high rate of digital illiteracy also extends to the providers, this limitation in the understanding of technologies has been a long-standing problem as many healthcare providers tend to not have the time or resources to spare learning or keeping up to date with the new advancements as the providers are always under high stress and a heavy patient load when compared to developed countries [9]. A study conducted in 2022 supports this claim as it found the medical doctor per 10 000 population is 9.66 in Iraq, which is much lower than our neighboring countries such as Iran, Jordan and Kuwait some of which have double the number [8, 22]. This barrier is also highlighted throughout the country in various forms due to a plethora of reasons such as language barriers, lack of training programs and lack of resources to allow for the practical application of such training programs and the overall lack of time and ability to balance a new system with the heavy load of patients that providers face on a daily basis [8, 9, 19].

The most prominent barrier is the limited availability of resources to build a culturally and practically compatible EHR system [9, 18]. While it is in no doubt that the Iraqi authorities had offered health system a certain priority, this priority has been a limit in and of itself due to the lack of or slowly degrading infrastructure due to the country’s experiences of conflicts and wars, which left the general infrastructure of healthcare and many other services destroyed [8, 11]. When compared to the state of care provided by other countries it can be seen how uniquely affected Iraq is in terms of not only the resources, but culturally, and infrastructurally as well. When comparing to Iran, for example, which had established a nationwide initiative that is still in use currently despite their conflicts [15, 16], and compared to the success of countries like the US, UK and Australia, we can see the stark difference in the willingness to change between cultures, let alone the availability of infrastructure and updated technologies that are well-maintained [7, 9, 11].

Furthermore, the lack of appropriate staff to build and maintain the system, and the overall negligence of the strong evidence provided by other countries that has implemented successful EHR, represents another major barrier [9, 19]. Additionally, the lack of upholding international standards in terms of cybersecurity, awareness and education decreases the trust and willingness to digitalize healthcare information [2, 4, 5, 8, 9, 18, 19, 21]. A study has highlighted that the main concerns regarding security arises from the inadequate information technology and lack of technical skills of staff due to suboptimal awareness in regards to appropriate usage of digital records, this in turn, directly raises the risk of external threats such as hacking and malware [19].

## ACTIONS FOR REFORM

It must be noted that many organizations have tried and have partially implemented digital recording systems within Iraq, but the fact still remains that the health sector is struggling to implement the technology nationwide [19]. It is imperative to acknowledge that the Kurdistan Region had integrated paper-based maternal and child healthcare files into the region’s health information system digitally. Despite its limited scope of effect, it had equipped policy makers with reliable information to recognize weaknesses and strengths and act accordingly. This pilot initiative had a considerable impact, which highlights the immeasurable potential benefits to gain if a nationwide electronic recording system is implemented [17, 19].

This overall inability to implement a standard highlights the need of an urgent multisectoral collaboration and good leadership that establishes an end goal with a general idea of what the sector needs, providing cloud-based security for privacy and easy access; having a good direction and blueprint may allow for the creation of a viable system [17, 19, 22]. Obstacles can be tackled by decreasing the difficulty and enhancing skill levels required to access and implement such a system through providing training courses, efficient financing of the projects, monetary incentives for applying such skills, and encouraging an environment that nurtures a generation of providers with the necessary operating skills to further advance our technological standard in healthcare within our developing country [2, 3, 8, 9, 13, 21, 23–26].

It is important to highlight, however, that the lack of infrastructure and resources may prove to be the cause of lack of incentive to pursue the establishment of foundations for nationwide digital health records [22]. Thus, we recommend, based on the evidence from our neighboring countries and most successful international health systems, that in the short-term, awareness must be increased and so the public can be educated in regards of the benefits and capabilities they may have access to if they support the implementation of digital records. Keeping in mind the cultural norms, we need to keep our people confident regarding the security of their information and update our confidentiality laws to further improve the trust and willingness of our population to use the system. Long term objectives should foster partnership between multiple stakeholders, local health authorities, medical academia and international partners to allow for the integration of digital health training programs in medical colleges, which will raise the competence of future healthcare workers to allow for a more efficient and easier implementation [7, 11, 17]. Repairing health system infrastructure must be the initial and the most prioritized step, as the state of primary health centers is in desperate need of revision and improvement, providing staff with the necessary knowledge and providing training to the healthcare providers in term of appropriate usage of electronic health records will allow a feasible and efficient implementation of digital records in our country [10, 20].

With the implementation of a nationwide digital record, the benefits do outweigh the potential risks, but it is appropriate to address such risks and potential drawbacks to appropriately prepare and establish countermeasures for such situations. Privacy issues and breaches of security are the most important potential risks as technology develops so do malicious sides that are capable of breaching secure systems [20]. Secondly, the potential of data corruption and system crashes due to electrical outages which are rather commonplace in a developing country such as Iraq, and last but not least, poor maintenance of equipment due to lack of or shortage of specialized staff as the demand would rapidly increase for specialized technicians for support and maintenance [11]. We are capable of addressing such issues through appropriate resource allocation, having funding for a specialized staff, allows for continuous maintenance of security systems and needed fixes in cases of system crashes or shutdowns. In regards to electrical shortages, appropriate resources can be allocated to provide health institutions with continuous electricity and backup batteries for system servers to prevent the loss of data [7, 11, 17].

## CONCLUSIONS

It is within medicine’s nature to continuously evolve and make use of all the information, data and technological advancements made to further enhance care. Digital health records have been a vital recent advancement. However, Iraq is struggling to establish such an important program, as the country has faced many conflicts and had lost its health infrastructure, which proves to be a major challenge alongside the digital illiteracy of both the public and healthcare providers. To overcome these barriers, governmental efforts must encourage the change through providing awareness, educational programs and resources as needed. Since the correct implementation of digital health records allows for a massive improvement in the standard care provided within Iraq. Medicine is an art that requires many tools, and as the tools and stage keep evolving, so must we.

## Data Availability

There are no new data associated with this article.
